# Infection by haemopathogens and tick infestation of sheep during summer season in Constantine region, Northeast Algeria

**DOI:** 10.1002/vms3.551

**Published:** 2021-06-17

**Authors:** Asma Amina Foughali, Mohamed Jedidi, Moktar Dhibi, Moez Mhadhbi, Limam Sassi, Ali Berber, Idir Bitam, Mohamed Gharbi

**Affiliations:** ^1^ Laboratoire de Biodiversité et Environnement: Interactions et Génomes Université des Sciences et de la Technologie Houari Boumédiène Algies Algeria; ^2^ Institut des Sciences Vétérinaires Université Saad Dahlab Blida Algeria; ^3^ Laboratoire de Parasitologie, Institution de la Recherche et de l'Enseignement Supérieur Agricoles & Univ. Manouba École Nationale de Médecine Vétérinaire de Sidi Thabet Sidi Thabet Tunisia; ^4^ Laboratoire des Biotechnologies Liées à la Reproduction Animale (LBRA) Université Blida 1 Blida Algeria; ^5^ École Supérieure en Sciences de l'Aliment et des Industries Agroalimentaires (ESSAIA), El Harrach, Alger Algeria

**Keywords:** Algeria, *Anaplasma*, *Babesia*, sheep, *Theileria*, tick

## Abstract

A study on tick infestation of 43 sheep with clinical symptoms of piroplasmosis and anaplasmosis was carried out during summer seasons of 2016 and 2017 in 34 sheep flocks from Beni Hamidene locality, district of Constantine, Northeast Algeria. Only animals with clinical symptoms of piroplasmosis and/or anaplasmosis were checked for tick infestation. Among the 43 examined sheep, 58 ± 15% were infested by ticks. A total of 185 adult ticks (100 males and 85 females) were collected from the 25 sheep. Two tick genera, *Rhipicephalus* and *Hyalomma* consisting of four species were collected, *Rhipicephalus bursa* was the most frequent tick (164/185; 88.6 ± 4.6%), followed by *Rhipicephalus sanguineus* sensu lato (16/185; 8.6 ± 4%), *Rhipicephalus* (*Boophilus*) *annulatus* (4/185; 2.2 ± 2.1%) and *Hyalomma scupense* (1/185; 0.5 ± 1%). Mean overall tick infestation intensity was 7.4. Mixed infestations with two tick species were found in 10 sheep (23.3 ± 12.6%). All farmers (34/34) used ivermectin, and only 11% of them used acaricide to control ticks. The majority of *R. bursa* ticks (87/185; 45 ± 7%) were located on the ears 37/185; 20 ± 6%) and the testicles (50/185; 27 ± 6%). Giemsa‐stained blood smears examination showed the presence of three haemopathogens: *Anaplasma* spp. (19/43; 44 ± 15%); *Babesia* spp. (6/43; 14 ± 10%) and *Theileria* spp. (1/43; 2 ± 4%). These results showed the presence of several low infestation burdens by ticks transmitting three haemopathogens.

## Graphical abstract

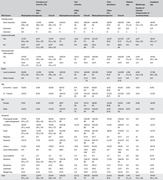

## 1 INTRODUCTION

Ticks are obligate haematophagous ectoparasites that can transmit several pathogens to humans and animals (Brites‐Neto et al., 2015; Dabaja et al., 2017; Merino et al., 2013; Peter et al., 2005). Among these pathogens, *Babesia*, *Theileria* and *Anaplasma* infections represent in sheep a real health problem in several regions of the world (Aydin et al., 2013; Iqbal et al., 2013; Li et al., 2020; Ozubek & Aktas, 2017). The epidemiology of these infections is dramatically influenced by abiotic factors, mainly temperature and humidity (Borşan et al., 2020; Estrada‐Pena, 2009). Ticks cause significant economic losses to farm animal industry, they represent in some regions in the world the main constraint to the development of ruminant's breeding (Aydin et al., 2015; Jongejan & Uilenberg, 2004).

In Adrar (southwest of Algeria), 784 ticks were collected from slaughtered sheep, they belonged to two genera and seven species: *Hyalomma impeltatum* (572/784; 75.2%), *H*. *marginatum* (19/784; 2.5%), *H. dromedarii* (9/784; 1.2%), *H. scupense* (2/784; 0.3%), *R. sanguineus* (148; 19.4%), *R. guilhoni* (10/784; 1.3%) and *R. evertsi* (1/784; 0.1%) (Bouhous et al., 2011).

In Algeria, ovine babesiosis is caused by *Babesia ovis* and *Babesia motasi* (Aouadi et al., 2017; Darghouth, 2004) inducing in clinically patent infections fever, anorexia, apathy, anaemia, icterus, haemoglobinuria with high morbidity and lethality rates (Esmaeilnejad et al., 2014; Ijaz et al., 2013; Sevinc et al., 2013, 2018; Yeruham et al., 1998).

Ovine anaplasmosis represents a major health problem in sheep flocks in several tropical and subtropical regions (Shi et al., 2020). This rickettsia is transmitted by ticks and mechanically by serval haematophagous insects. Symptoms are not specific: fever, apathy, anorexia, anaemia, icterus, nasal discharge, weight loss, hypogalactia and abortion (Gharbi et al., 2015; Tibbitts et al., 1992; Yasini et al., 2012).

In North Africa, several studies reported the presence of *Theileria* spp. in sheep specially *Theileria lestoquardi* and *Theileria ovis* (Al‐hosary et al., 2021; Hussein et al., 2017; Rjeibi et al., 2014). *Theileria lestoquardi* causes malignant ovine theileriosis (MOT) which is by far more pathogenic than *T. ovis* (Hassan et al., 2019; Yaghfoori et al., 2016). The clinical symptoms of MOT are similar to tropical theileriosis in cattle (*Theileria annulata* infection) and can cause death in severe cases (Ge et al., 2012).

We aimed herein to study the tick infestation during the tick season in sheep with clinical episodes or heamopathogen infections in Beni Hamidene locality, district of Constantine, Northeast Algeria.

## 2 MATERIALS AND METHODS

### 2.1 Study area and period

The present survey was carried out in Beni Hamidene locality (36°30′ N; 6°31′ E), (district of Constantine, Northeast Algeria) during 2017 and 2018 summer seasons, between early June and late August. Beni Hamidene locality has an altitude that varies between 300 and 1364 m and an area of 131 km^2^. The climate of the region is sub‐humid and semi‐arid with a mean annual precipitation of 761 mm (Direction des Services Agricoles de la Wilaya de Constantine, 2015). In the district of Constantine, the sheep population was estimated to 171,187 sheep, among them 108,985 ewes (Direction des Services Agricoles de la Wilaya de Constantine, 2016).

### 2.2 Farms and sheep

The present survey was performed in 34 sheep flocks containing a total number of 3200 sheep with a mean sheep population of 74 (range: 14–150). The majority of sampled animals were maintained under semi‐intensive (91 ± 9%; 31/34;), followed by extensive (6 ± 8%; 2/34) and intensive systems (3 ± 6%; 1/34).

Sheep were housed with other domestic animals (goats, cattle, dogs and chickens) in 77 ± 14% (26/34) of the farms. The majority of sheep shelters had litters of bad quality (24/34; 71 ± 15%), and 88.2 ± 10.8% (30/34) of barns’ walls were cracked. The majority of farms were specialised in meat production and reproduction of ewes (26/34; 77 ± 14%), 5 (15%) were fattening farms, and three (9%) were specialised in ewes’ reproduction.

A total number of 43 (1.3%) sheep showed clinical symptoms (anaemia, icterus, anorexia, fever, asthenia and lymph nodes enlargement) were included in the present survey. These animals were aged between 3 months and 7 years (mean age: 1.7 years) and consisted of 20 females and 23 males (sex ratio M:F = 1.15). Animals belonged to two breeds, local crossbred (38/43; 88 ± 10%) and other breeds (5/43; 12 ± 10%), namely, Ouled Djellal, Rembi and Berbere.

The farmers were questioned about the symptoms expressed by their animals and the use of acaricides. Animal owners were questioned about the application of acaricides on their animals, and a verbal consent was obtained from each farmer. Sheep were clinically examined; their ages were estimated by examining their teeth.

### 2.3 Blood samples collection

Blood samples were collected from the ear capillaries of each sheep using a disposable scalpel; blood smears were immediately made in the farms, fixed in the methanol for 3 min and Giemsa‐stained.

Blood smears were examined under a microscope with immersion oil at x1000 magnification.

### 2.4 Ticks collection and identification

All ticks fixed on the examined sheep were collected and stored in identified tubes containing 70% ethanol. Ticks were identified under a stereomicroscope according to the key of Walker et al. (2014).

### 2.5 Parasitological indictors and statistical analyses

Three parasitological indicators were estimated (Bush et al., 1997) as follows:

$$\begin{array}{ccc} \mathrm{Infestation}\;\mathrm{prevalence}\;\left(\%\right) & = & 100\times \mathrm{Number}\;\mathrm{of}\;\mathrm{infested}\\ & & \mathrm{sheep}/\mathrm{number}\;\mathrm{of}\;\mathrm{examined}\\ & & \mathrm{sheep}\\ \mathrm{Infestation}\;\mathrm{intensity} & = & \mathrm{Number}\;\mathrm{of}\;\mathrm{collected}\;\mathrm{ticks}/\\ & & \mathrm{number}\;\mathrm{of}\;\mathrm{infested}\;\mathrm{sheep}\\ \mathrm{Ticks}\;\mathrm{abundance} & = & \mathrm{Number}\;\mathrm{of}\;\mathrm{collected}\;\mathrm{ticks}/\\ & & \mathrm{number}\;\mathrm{of}\;\mathrm{examined}\;\mathrm{sheep} \end{array}$$

The infection intensity by haemopathogens was estimated by calculating the number of infected‐red blood cells in 50 microscope fields (on average, each microscopic field contains 350 red blood cells).

### 2.6 Treatments

All sheep with clinical symptoms of anaplasmosis and/or piroplasmosis were treated. Oxytetracycline (Longicine^®^, Vetoquinol, France) was injected at the dose of 20 mg/kg to animals suffering from anaplasmosis. Sheep presenting anaemia (33/43; 77%) were treated with an anti‐anaemic drug (Hematofos B12^®^, Agrovet Market, Peru) at the dose of 5 ml/sheep. Diminazene (FA.TRY.BANIL^®^, Fatro, Italy) was injected to sheep with piroplasmosis clinical signs at the dose of 3.5 mg/kg. Sheep with fever (32/43; 74%) received meloxicam (Melovem^®^ 20 mg/ml, Dopharma, Netherlands) at the dose of 0.5 mg/kg.

Half a sachet of Rumicen Poudre Complex (Cenavisa, Spain) was administered orally to two sheep with digestive conditions. Erythromycin (Erythrocine^®^, Ceva, France) was injected intramuscularly at the dose of 10 mg/kg every 12 h for 3 days to treat lameness. Oxytetracycline Wound Spray (Terramycine, Zoetis, USA) was applied on the animals’ toes.

## 3 RESULTS

Among the questioned farmers, only four (4/34; 12 ± 11%) used acaricides to control ticks (Phoxim, Sebacil^®^ 50%, Bayer, Germany). All of them (34/34) used ivermectin (Biomectin 1%^®^, Vetoquinol, France) and thought that it was effective against ticks.

### 3.1 Parasitological indicators

**FIGURE 1:**
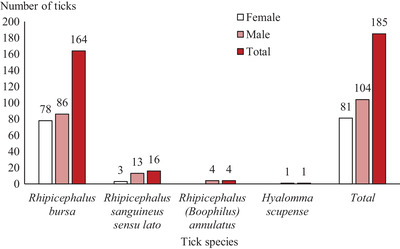
Number of ticks collected from sheep, in the locality of Beni Hamidene (district of Constantine, Algeria)

More than half of sheep were infested by at least one tick (25/43; 58 ± 15%). A total of 185 adult ticks were collected, consisting of 100 males and 85 females (sex ratio M: F = 1.18). Collected ticks belonged to two genera: *Rhipicephalus* and *Hyalomma* and four species: *Rhipicephalus bursa* (164/185; 88.6 ± 4.6%), *Rhipicephalus sanguineus* sensu lato (16/185; 8.6 ± 4 %), *Rhipicephalus* (*Boophilus*) *annulatus* (4/185; 2.2 ± 2.1%) and *Hyalomma scupense* (1/185; 0.5 ± 1%) (Figure 1).

Overall mean tick infestation intensity was 7.4. Mean infestation abundance was 4.3. Co‐infestation by two tick species was found in 10 sheep (23 ± 13%) of the 43 examined sheep.

**FIGURE 2:**
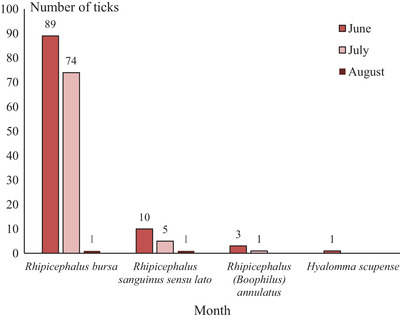
Monthly evolution of the number of ticks infesting sheep in Beni Hamidene locality (district of Constantine, Northeast Algeria)

The activity peak of *R*. *bursa* occurred in June, when more than half of them were collected (89/164; 54.3 ± 7.6%) (Figure 2). A total number of 164 *R. bursa* ticks were collected, more than half of them (86/164; 52.4 ± 7.6%) were male *R. bursa*.

### 3.2 Infestation by ticks according to anatomical regions of sheep

**FIGURE 3:**
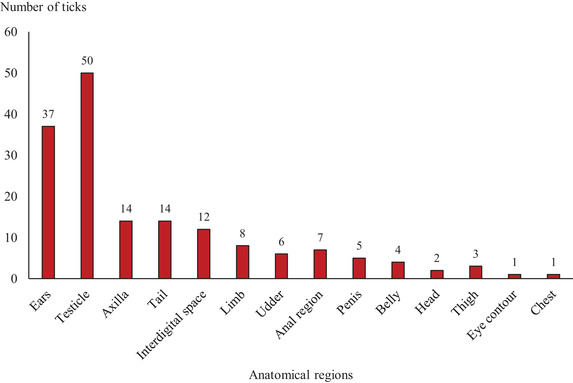
Anatomical localization of *Rhipicephalus bursa* on sheep from the locality of Beni Hamidene (district of Constantine, Northeast Algeria)

Almost half of *R. bursa* collected ticks (87/185; 45 ± 7%) were found on the testicles (50/185; 27 ± 6%), followed by ears (37/185; 20 ± 6%) (Figure 3). All *R. sanguineus* sensu lato (16/16) tick specimens were collected from the ears.

### 3.3 Haemopathogens

Among the 34 visited sheep flocks, 18 (53 ± 17%) contained at least one sheep infected by at least one haemopathogen, 12 (35 ± 16%) sheep flocks contained only sheep infected by one haemopathogen, and six (18 ± 13%) sheep flocks contained only co‐infected sheep. Among the 43 sheep with clinical symptoms, almost half of them (20/43; 46 ± 15%) were infected by at least one haemopathogen.

Among the 25 infested sheep by ticks, 12, 2 and 1 animal were infected by *Anaplasma* spp., *Babesia* spp. and *Theileria* spp., respectively (Table 1).

**TABLE 1:** Characteristic of farms, infested sheep by ticks and relation between symptoms and infestation by ticks in Beni Hamidene commune (district of Constantine, Algeria)

		Prevalence of ticks (% ± SE)			Tick intensity			Ticks abundance		*Theileria* spp.	*Babesia* spp.	*Anaplasma* spp.
Risk factors	*Rhipicephalus bursa*	Other tick species	Overall	*Rhipicephalus bursa*	Other tick species	Overall	*Rhipicephalus bursa*	Other tick species	Overall		Number of infected sheep/number of examined sheep	
*Farming system*												
Semi‐intensive	23/40 (58 ± 15)	11/40 (28 ± 14)	24/40 (60 ± 15)	164/23 (7)	18/11 (2)	182/24 (8)	164/40 (4)	18/40 (0.5)	182/40 (5)	1/40 (3 ± 5)	6/40 (15 ± 11)	18/40 (45 ± 15)
Extensive	0/2	1/2	1/2	0	3/1	3/1	0	3/2	3/2	0/2	2	1/2
Intensive	0/1	0	0/1	0	0	0	0	0	0	1	1	1
*Cracks in the walls*												
Yes	17/37 (46 ± 16)	8/37 (22 ± 13)	19/37 (51 ± 16)	121/17 (7)	16/8 (2)	137/19 (7)	121/37 (3)	16/37 (0.4)	137/37 (3.7)	1/37 (3 ± 5)	6/37 (16 ± 12)	18/37 (49 ± 17
No	6/37 (16 ± 12)	4/37 (11 ± 10)	6/37 (16 ± 12)	43/6	5/4	48/6 (8)	43/6 (7)	5/6	48/6 (8)	0/6	0/6	1/6
*Promiscuity with others species*												
Yes	19/34 (56 ± 17)	11/34 (32 ± 16)	21/34 (62 ± 16)	150/19 (8)	16/11 (2)	166/21 (8)	150/34 (4)	16/34 (0.5)	166/34 (5)	1/34 (3 ± 6)	5/34 (15 ± 12)	14/34 (41 ± 16)
No	4/34 (12 ± 11)	1/34 (3 ± 6)	4/34 (12 ± 11)	14/4	5/1	19/4	14/9	5/9	19/9	0/9	1/9	5/9
*Breed*												
Local crossbred	20/38 (53 ± 16)	11/38 (29 ± 14)	22/38 (58 ± 16)	145/20 (7)	19/11 (2)	164/22 (8)	145/38 (4)	19/38 (0.5)	164/38 (4)	1/38 (3 ± 5)	6/38 (16 ± 12)	17/38 (45 ± 16)
Other breeds	3/5	1/5	3/5	19/3	2/1	21/3 (7)	19/5	2/5	21/5 (4.2)	0/5	0/5	2/5
*Age*												
(3 months ‐ 1 year)	10/20	4/20	10/20	60/10 (6)	4/4 (1)	64/10 (6)	60/20 (3)	4/20 (0.2)	64/20 (3)	0/20	4/20	8/20
(1 ‐ 7 years)	13/23	8/23	15/23	104/13 (8)	17/8 (2)	121/15 (8)	104/23 (5)	17/23 (0.7)	121/23 (5)	1/23	2/23	11/23
*Sex*												
Female	9/20	6/20	11/20	38/9 (4)	15/6 (3)	53/11 (5)	38/20 (2)	15/20 (0.8)	53/20 (3)	1/20	2/20	10/20
Male	14/23	6/23	14/23	126/14 (9)	6/6 (1)	132/14 (9)	126/23 (6)	6/23 (0.3)	132/23 (6)	0/23	4/23	9/23
Symptom												
Prescapular lymph node enlargement	19/32 (59 ± 17)	9/32 (28 ± 16)	20/32 (63 ± 17)	143/19 (8)	13/9	156/20 (8)	143/32 (4)	13/32	156/32 (5)	0/1	4/6	13/19
Anorexia	18/35 (51 ± 16.6)	10/35 (29 ± 15)	19/35 (54 ± 17)	145/18 (8)	15/10	160/19 (8)	145/35 (4)	15/35	160/35 (5)	1/1	6/6	13/19
Fever	20/35 (57 ± 16)	11/35 (31 ± 15)	22/35 (63 ± 16)	161/20 (8)	18/11	179/22 (8)	161/35 (5)	18/35	179/35 (5)	1/1	5/6	14/19
Petechia	3/7	2/7	4/7	25/3	2/2	27/4	25/7	2/7	27/7	1/1	/6	3/19
Anaemia	13/19	7/19	14/19	105/13 (8)	11/7	116/14 (8)	105/19 (6)	11/19	116/19 (6)	0/1	4/6	10/19
Icterus	11/23	6/23	13/23	45/11	14/6	59/13	45/23	14/23	59/23	1/1	1/6	10/19
Local inflammation	4/4	2/4	4/4	68/4	2/2	70/4 (18)	68/4 (17)	2/4	70/4 (18)	0/1	1/6	2/19
Local oedema	1/1	1/1	1/1	4/1	1/1	5/1	4/1	1/1	5/1	0/1	0/6	0/19
Asthenia	18/33 (55 ± 17)	8/33 (24 ± 15)	19/33 (58 ± 17)	135/18 (8)	11/8	146/19 (8)	135/33 (4)	11/33	146/33 (4)	0/1	5/6	15/19
Dyspnoea	6/11	4/11	6/11	32/6	5/4	37/6	32/11	5/11	37/11	0/1	2/6	3/19
Lameness	2/2	1/2	2/2	10/2	1/1	11/2	10/2	1/2	11/2	0/1	1/6	1/19
Weight loss	9/13	6/13	9/13	47/9	7/6	54/9	47/13	7/13	54/13	0/1	2/6	4/19
*Total*	23/43 (54 ± 15)	12/43 (28 ± 13)	25/43 (58 ± 15)	164/23 (7)	21/12 (2)	185/25 (7)	164/43 (4)	21/43 (0.5)	185/43 (4)	1/43 (2 ± 5)	6/43 (14 ± 10)	19/43 (44 ± 15)

Among the 19 *Anaplasma*‐infected sheep, five were co‐infected by *Anaplasma* spp. and *Babesia* spp., and one was co‐infected by *Anaplasma* spp. and *Theileria* spp.

The infection intensity in the sheep infected by *Theileria* sp. was low (0.1. 10^–3^). It varied between 0.3. 10^–3^ and 9. 10^–3^ for *Anaplasma* spp. and from 0.06. 10^–3^ and 3. 10^–3^ for *Babesia* spp. (Table 1).

The majority of sheep with clinical symptoms showed fever (35/43; 81 ± 16), anorexia (35/43; 81 ± 12%) followed by prescapular lymph node enlargement (32/43; 74 ± 13%). More than half of them developed icterus (23/43; 53 ± 15). The highest number of ticks were collected from sheep with fever (179/185; 97 ± 3), almost all of them were *R. bursa* (161/164; 98 ± 2). Lameness in two sheep was due to ticks fixed in the interdigital space. Petechia (eyes or internal face of the tail) were observed in only 16% (7/43).

### 3.4 Lethality

Among the 43 sheep suffering from piroplasmosis and/or anaplasmosis 4 (9 ± 9%) died just after treatment. Two dead sheep were co‐infected by *Anaplasma* spp. and *Babesia* spp. The two others died but the blood smears microscopic examination did not show any haemopathogen.

## 4 DISCUSSION

In the present study, the overall infestation prevalence by ticks was estimated to 58%, it was higher to the prevalence reported in Adrar (Southwest Algeria) by Bouhous et al. (2011) (29.7%), and in Tunisia by Rjeibi et al. (2015) (20.4%). The present work was performed on diseased sheep, while, the two previous authors worked on apparently healthy sheep. Only 12% of the questioned farmers use acaricide, this could be explained by two main reasons: the high cost of acaricides and the low awareness of the sheep owners about the vector role of ticks.

*Rhipicephalus bursa* represented 88% of collected ticks; the abiotic factors in Beni Hamidene are suitable for this species. The proportion of this species is higher than reported by Elati et al. (2018) (4/722; 1%), in Tunisia. This species is the most frequent sheep tick in northern Algeria; it is also the most important *B. ovis* vector (Andersson et al., 2017; Erster et al., 2016; Esmaeilnejad et al., 2014).

In Northeast Algeria, Leulmi et al. (2016) reported in Taref that, among the 529 collected ticks,72 were *R. bursa*. Abdelkadir et al. (2019) reported in Sidi Bel Abbes and Saida (Northwest of Algeria) that 59 of the 70 engorged or partially engorged ticks were *R. Bursa*.

*Rhipicephalus sanguineus* sensu lato represented only 8.6 % of collected ticks; this tick has the capacity to adapt to several hosts. However, *R. sanguineus* was the most frequent tick in Iran (Dehaghi et al., 2011) (36.37%), in Greece, Chaligiannis et al. (2018) reported that only one of 64 collected ticks from sheep was *R*. *sanguineus* sensu lato.

Few collected ticks were *Rhipicephalus* (*Boophilus) annulatus* (4/185; 2.2%). In northeast Algeria, Sadeddine et al. (2020) reported that, among 37 collected ticks from sheep, 14 were *Rh*. (*Boophilus*) *annulatus*.

Only one (0.5%) *Hy. scupense* ticks were collected form sheep; this species infests mainly cattle (Gharbi et al., 2013). The presence of *H. scupense* could be explained by the presence of cattle in 77% of the visited farms. In Algeria, Sadeddine et al. (2020), collected one *H. scupense* specimen from sheep out of 37 collected ticks.

Only adult ticks were collected, the absence of immature instars could be explained by the fact that the work was carried out in summer, generally only adults are present (Boulkaboul, 2003; Gharbi & Darghouth, 2014; Laamari et al., 2012).

*Rhipicephalus bursa* species was collected in different anatomical parts, particularly on the ears and testicles (87/164). The ears are in contact with vegetation when animals graze and are easily accessible to ticks. However, Papadopoulos et al. (1996) reported that anus, vulva, udder and scrotum are the attachment sites of *R. bursa*.

Mean overall infestation intensity was 7.4, it was higher than reported in Tunisia (Rjeibi et al., 2015) (2.41).

Giemsa‐stained blood smear was used to confirm the clinical suspicion. This technique is used in sick animals since they have relatively high parasitaemia (Altay et al., 2012). Out of the 43 sampled sheep, 47% were infected by at least one haemopathogen.

*Anaplasma* spp. was the most frequent haemopathogen (12/25) in the 25 tick‐infested sheep. *Anaplasma* is transmitted mechanically by several tick species. In Algeria, *Anaplasma ovis* was detected in sheep by the quantitative real‐time PCR (qPCR) in the northeast of Algeria (Aouadi et al., 2017; Sadeddine et al., 2020). *Anaplasma ovis* was identified in sheep in Senegal (55.9%) (Dahmani et al., 2019). A study on sheep anaplasmosis was carried out in Kairouan (central Tunisia, Kairouan); of the 8049 sheep examined by Giemsa‐stained blood smears, 282 were infected by *A. ovis* (Gharbi et al., 2015). Using PCR, Belkahia et al. (2014) reported that in Tunisia, 70.1% (143/204) of apparently healthy sheep were infected by *A. ovis*. In Sudan, Lee et al. (2018) reported that *A. ovis* was the most prevalent pathogen (52/62; 83.9%).

*Babesia* spp. was found in 14% of the examined sheep (6/43). This rate is higher than that reported by Kage et al. (2019) (8/225; 3.55%) who used Giemsa‐stained blood smears from both suspected and healthy sheep in Karnataka state, India. However, in our study, we worked only on sheep with clinical symptoms of anaplasmosis and/or piroplasmosis and the sampled animals was low. The prevalence was lower than the estimation reported by Naderi et al. (2017) in Iran (38/306; 9.9%) who worked on babesiosis‐suspected sheep.

Only one sheep (2%) was infected by *Theileria* spp. This rate is similar to that reported by Rjeibi et al. (2014) in Tunisia (2/166) by blood smears examination. However, G. R. Razmi et al. (2006) reported a rate of 11.9% (100/840) in South Khorasan province (Iran) using microscopic examination and concluded that *Theileria* infection was spreading in this region. In Turkey, the reverse line blotting (RLB) method detected that 28.9% (37/128) of apparently healthy sheep were infected by *Theileria* spp. (Altay et al., 2012). In Kenya, polymerase chain reaction revealed that 51.3% (39/76) of sheep were infected by *Theileria* spp. (Ringo et al., 2019). This could be explained by the diagnostic methods; PCR, qPCR and RLB are more sensitive and specific than Giemsa‐stained blood smears (Rjeibi et al., 2014; Shayan & Rahbari, 2005; Theodoropoulos et al., 2006; Wang et al., 2015). The majority of sheep with clinical symptoms of piroplasmosis and/or anaplasmosis (32/43; 74 ± 13) showed prescapular lymph node enlargement. Lymph node enlargement is observed in animals clinically infected by *T. lestoquardi*, which is transmitted mainly by *Hyalomma anatolicum* (G. R. Razmi et al., 2003; Taha & Elhussein, 2010). *Rhipicephalus turanicus* and *H. impeltatum* are suspected to be potential vectors of *T. lestoquardi* (El‐Azazy et al., 2001; G. Razmi et al., 2013).

Further studies are needed to identify the tick fauna infesting sheep in order to implement the correct tick‐control strategy and also to identify the species of haemopathogens infecting sheep.

## CONFLICT OF INTEREST

All the authors declare no conflict of interest with the work presented here.

## AUTHOR CONTRIBUTIONS

Asma Amina Foughali: Conceptualization, Data curation, Formal analysis, Investigation, Methodology, Software, Visualization, Writing‐review & editing. Mohamed JEDIDI: Validation, Visualization. Mokhtar Dhibi: Data curation, Visualization. Moez MHADHBI: Visualization. Limam Sassi: Validation. Ali Berber: Project administration. Idir Bitam: Project administration. Mohamed Gharbi: Data curation, Formal analysis, Methodology, Software, Validation, Writing‐review & editing.

### PEER REVIEW

The peer review history for this article is available at https://publons.com/publon/10.1002/vms3.551.

## Data Availability

The datasets generated during the current study are available from the corresponding author on reasonable request.
